# Cell-free DNA promoter hypermethylation in plasma as a predictive marker for survival of patients with pancreatic adenocarcinoma

**DOI:** 10.18632/oncotarget.21397

**Published:** 2017-09-30

**Authors:** Stine Dam Henriksen, Poul Henning Madsen, Anders Christian Larsen, Martin Berg Johansen, Inge Søkilde Pedersen, Henrik Krarup, Ole Thorlacius-Ussing

**Affiliations:** ^1^ Department of Gastrointestinal Surgery, Aalborg University Hospital, Aalborg, Denmark; ^2^ Department of General Surgery, Hospital of Vendsyssel, Hjørring, Denmark; ^3^ Department of Clinical Medicine, Aalborg University, Aalborg, Denmark; ^4^ Clinical Cancer Research Center, Aalborg University Hospital, Aalborg, Denmark; ^5^ Section of Molecular Diagnostics, Clinical Biochemistry, Aalborg University Hospital, Aalborg, Denmark; ^6^ Unit of Clinical Biostatistics and Bioinformatics, Aalborg University Hospital, Aalborg, Denmark

**Keywords:** biomarker, epigenetics, methylation, pancreatic cancer, prognosis

## Abstract

**Introduction:**

Few prognostic biomarkers are available for pancreatic cancer. The aim of this study is to examine the correlation between the survival of pancreatic adenocarcinoma patients and hypermethylated genes in plasma-derived cell-free DNA.

**Methods:**

Consecutive patients with pancreatic adenocarcinoma were prospectively included and staged according to the TNM classification. Methylation-specific PCR of 28 genes was conducted. A survival prediction model independent of cancer stage and stage-specific survival prediction models were developed by multivariable Cox regression analysis using backward stepwise selection.

**Results:**

Ninety-five patients with pancreatic adenocarcinoma were included. Patients with more than 10 hypermethylated genes had a HR of 2.03 (95% CI; 1.15-3.57) compared to patients with fewer hypermethylated genes. Three survival prediction models were developed: Total group; (American Society of Anesthesiologists score (ASA)=3, *GSTP1*, *SFRP2*, *BNC1*, *SFRP1*, *TFPI2,* and *WNT5A*) Risk groups 2, 3 and 4 had a HR of 2.65 (95% CI; 1.24-5.66), 4.34 (95% CI; 1.98-9.51) and 21.19 (95% CI; 8.61-52.15), respectively, compared to risk group 1. Stage I-II; (ASA=3, *SFRP2,* and *MESTv2*) Risk groups 2, 3 and 4 had a HR of 4.83 (95% CI; 2.01-11.57), 9.12 (95% CI; 2.18-38.25) and 70.90 (95% CI; 12.63-397.96), respectively, compared to risk group 1. Stage IV; (*BMP3*, *NPTX2*, *SFRP1*, and *MGMT*) Risk group 2 had a HR of 5.23 (95% CI; 2.13-12.82) compared to risk group 1.

**Conclusion:**

Prediction models based on cell-free DNA hypermethylation stratified pancreatic adenocarcinoma patients into risk groups according to survival. The models have the potential to work as prognostic biomarkers. However, further validation of the results is required to substantiate the findings.

## INTRODUCTION

Pancreatic cancer is the 4^th^ leading cause of cancer death in developed countries [1], with a five-year survival rate between 5-7% [2]. The current standard for determining patient prognosis for pancreatic cancer is the extent of the disease, defined by the primary tumor (T), lymph node (N) and distant metastasis (M) staging system [3]. Patients undergoing intended curative treatment have a five-year survival rate of approximately 14%, whereas only 1% of patients with distant metastases are alive after five years [2]. Furthermore, there is a significant range in survival time within individual clinical stage. This intra-stage variance might reflect different tumor biology, caused by various gene expression profiles [4].

There are few prognostic markers available for pancreatic cancer. CA-19-9, which is not suitable as a diagnostic marker for pancreatic cancer, has been suggested as a prognostic marker [5], [6]. However, the utility of CA-19-9 is limited, as 10% of the population lack the ability to express CA-19-9 [5], [6]. Performance status is also used as a prognostic factor for pancreatic cancer, as patients with a poor performance status are less likely to overcome extensive surgery or benefit from intensified chemotherapy regimens [7], [8].

It would be of great benefit to patients if additional markers for prognosis were available, to identify patients with more aggressive tumor biology upfront. It would optimize therapeutic decision-making and promote individualized therapy.

During the development of pancreatic cancer, a variety of genetic and epigenetic changes occurs. Genetic alterations change the DNA sequence, whereas epigenetic modifications change the DNA conformation and the chromatin structure, and consequently, the gene expression changes. DNA hypermethylation is an epigenetic mechanism, where a methyl (CH_3_) residue is added to cytosines preceding guanosines (CpGs) [9–12]. Hypermethylation in the promoter region is one of the mechanisms that can lead to inactivation of tumor suppressor genes associated with carcinogenesis [9], [10], [13], [14].

In different types of cancer, DNA hypermethylation has been reported to have prognostic value and to be an independent predictor of survival [15–18]. We have previously performed a literature review regarding genes aberrantly methylated in pancreatic cancer [19]. Furthermore, we have shown that hypermethylated genes in plasma is useful as a diagnostic marker for pancreatic adenocarcinoma [20].

The aim of this study is to examine the correlation between survival of patients with pancreatic adenocarcinoma and hypermethylated genes in plasma-derived cell-free DNA, both as a general predictive marker for survival and as stage-specific predictive survival markers.

## RESULTS

In this study, 95 patients with pancreatic adenocarcinoma were included. Table 1 lists the baseline characteristics of the patients.

**Table 1:** Baseline characteristics of patients with pancreatic adenocarcinoma (N=95)

Stage	I (Ia+Ib)		II (IIa+IIb)		III		IV	
N	11		29		13		42	
Age (mean) (SD)	70	(10.81)	67	(8.21)	65	(8.25)	65	(9.21)
Sex (men:women)	6:5		19:10		10:3		22:20	
ASA 1 (n) (%)	4	36%	14	48%	8	62%	0	0%
ASA 2 (n) (%)	4	36%	11	38%	3	23%	18	43%
ASA 3 (n) (%)	3	27%	4	14%	2	15%	12	29%
Intendent curative surgery (n) (%)	5	45%	24	83%	4	31%	1	2%
Preoperative chemotherapy (n) (%)	-	-	3	10%	-	-	-	-
Palliative chemotherapy (n) (%)	3	27%	9	31%	10	77%	27	64%

SD: Standard deviation

ASA: American Society of Anesthesiologists score.

Note: Stage is in accordance with The American Joint Committee on Cancer (AJCC) stage classification.

### Survival analyses

#### Survival analyses according to staging

**Figure 1:**
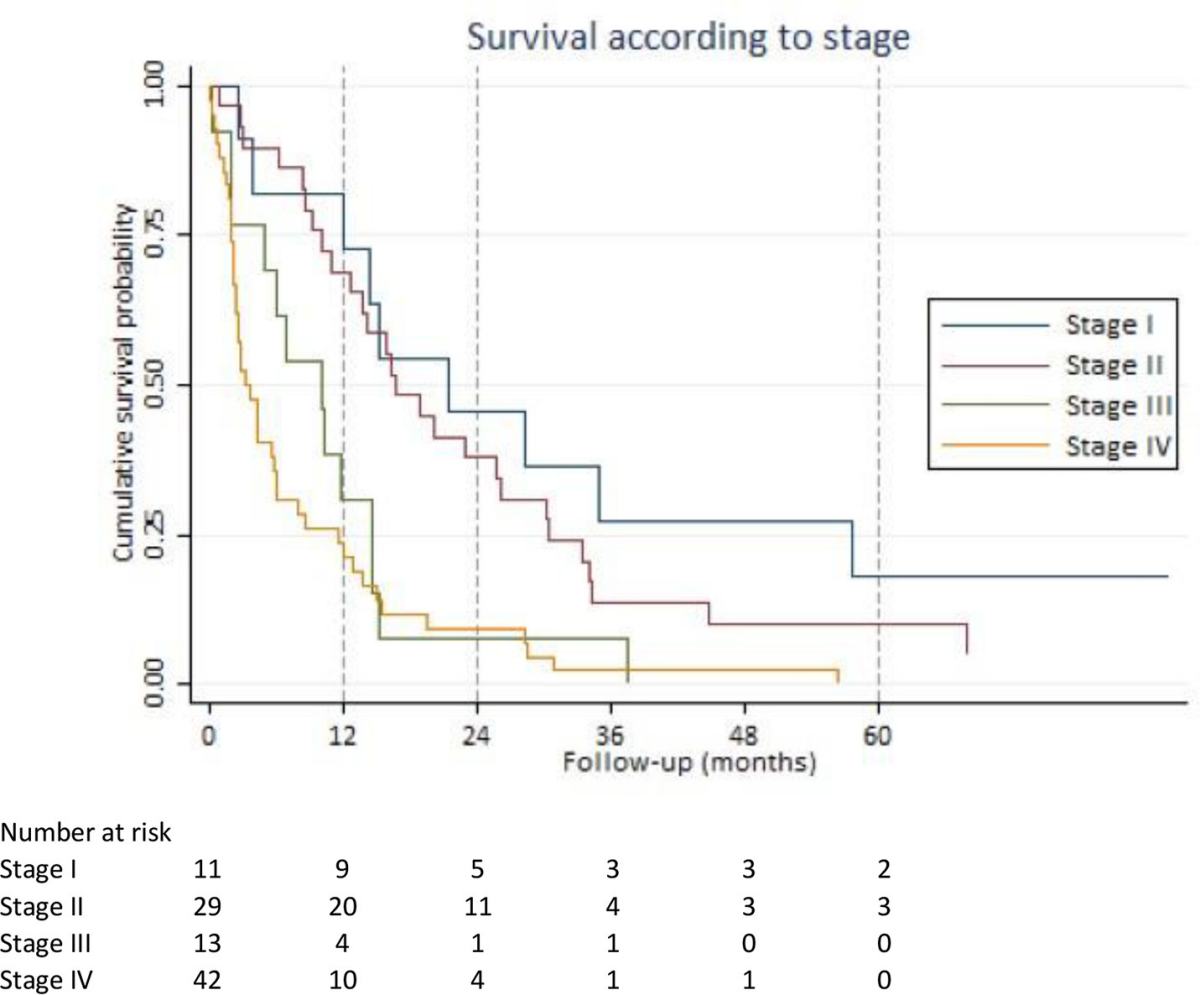
Survival according to stage Kaplan-Meier survival estimates based on American Joint Committee on Cancer stage classification.

The Kaplan-Meier curves in Figure 1 show survival according to pancreatic cancer staging.

### Survival analysis according to the total number of hypermethylated genes

**Figure 2:**
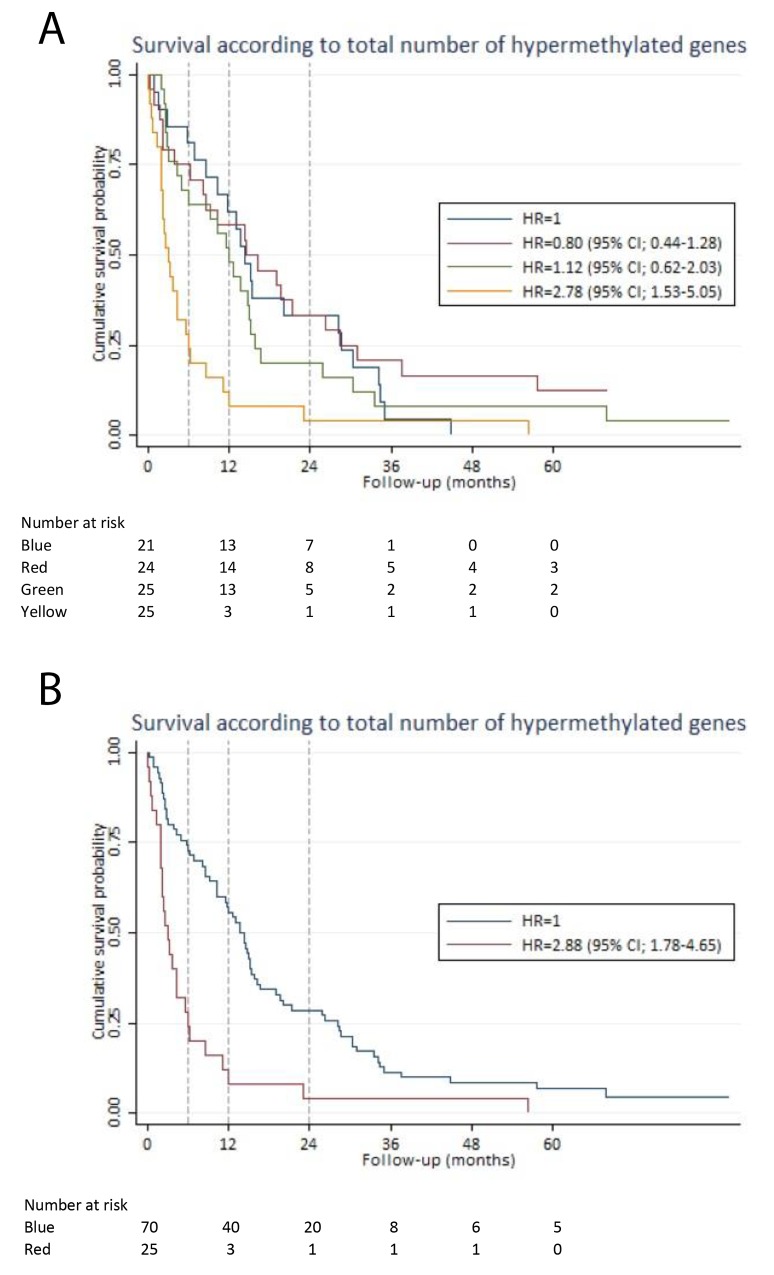
Survival according to the total number of hypermethylated genes For each patient, the total number of hypermethylated genes was calculated. Based on that calculation, the patients were divided into quartiles. The Kaplan-Meier curves illustrate the survival estimates according to the total number of hypermethylated genes in plasma-derived cell-free DNA. **(A)** Blue line: 1^st^ quartile (1-5 hypermethylated genes). Red line: 2^nd^ quartile (6-7 hypermethylated genes). Green line: 3^rd^ quartile (8-10 hypermethylated genes). Yellow line: 4^th^ quartile (>10 hypermethylated genes). There was no significant difference in HR between the 1^st^, 2^nd^ and 3^rd^ quartile. However, the 4^th^ quartile had a HR of 2.78 (95% CI; 1.53-5.05). **(B)** Blue line: 1^st^ quartile, 2^nd^ quartile and 3^rd^ quartile (1-10 hypermethylated genes) were combined as the survival estimates were identical for the first three quartiles (see Figure 2A). Red line: 4^th^ quartile (>10 hypermethylated genes) The 4^th^ quartile had a HR of 2.88 (95% CI; 1.78-4.65) compared to the combined group of the 1^st^, 2^nd^ and 3^rd^ quartiles.

Patients were divided into quartiles based on the total number of hypermethylated genes: 1^st^ quartile (1-5 hypermethylated genes), 2^nd^ quartile (6-7 hypermethylated genes), 3^rd^ quartile (8-10 hypermethylated genes) and 4^th^ quartile (11-20 hypermethylated genes). There was no significant difference in HR of the 1^st^, 2^nd^ and 3^rd^ quartiles. However, the 4^th^ quartile had a HR of 2.78 (95% CI; 1.53-5.05), which was significantly different (p-value < 0.001) from the 1^st^ quartile (Figure 2A). We combined the 1^st^, 2^nd^ and 3^rd^ quartiles (1-10 hypermethylated genes) and compared them to the 4^th^ quartile (more than 10 hypermethylated genes). In an analysis adjusted for staging and age, a HR of 2.03 (95% CI; 1.15-3.57) was found for patients with more than 10 hypermethylated genes. Patients with 0-10 hypermethylated genes had a better six-month, one-year and two-year survival (73% (95% CI; 61%-82%), 56% (95% CI; 43%-66%), and 28% (95% CI; 19%-39%)), compared to patients with more than 10 hypermethylated genes, who had a six-month, one-year and two-year survival of 28% (95% CI; 12%-46%), 12% (95% CI; 3%-28%), and 4% (95% CI; 0.3%-17) (Figure 2B).

### Prognostic prediction model development for the total group of patients

We first analyzed the total group of cancer patients, without taking into account the subsequent stage classification. The purpose was to develop a prognostic prediction model, usable prior to the final stage classification. By univariate screening, eight genes (*BNC1*, *GSTP1*, *MLH1*, *SFRP1*, *SEPT9v2*, *SST*, *TFPI2*, and *WNT5A*) yield a significant HR (Table 2). In addition, patients with an ASA score of three compared to an ASA score of one had a HR of 2.63 (95% CI; 1.49-4.63), and PS > 0 compared to PS = 0 was associated with a HR of 2.49 (95% CI; 1.61-3.84). The HRs for age and gender were insignificant.

**Table 2:** Hazard ratio for each gene based on univariate Cox regression analysis

*Gene*	*All stages (N = 95)*			*Stage I/II (N = 40)*			*Stage III (N = 13)*			*Stage IV (N = 42)*		
	*HR*	*P*	*95% CI*	*HR*	*P*	*95% CI*	*HR*	*P*	*95% CI*	*HR*	*P*	*95% CI*
*ALX4*	1.43	0.20	(0.83-2.47)	0.82	0.78	(0.19-3.43)	1.00	-	-	0.96	0.91	(0.50-1.86)
*APC*	0.99	0.97	(0.58-1.70)	0.88	0.76	(0.38-2.01)	0.40	0.21	(0.09-1.69)	1.34	0.51	(0.56-3.19)
*BMP3*	1.41	0.13	(0.91-2.18)	0.80	0.59	(0.37-1.77)	0.71	0.58	(0.21-2.41)	**3.21**	**0.00**	**(1.58-6.53)**
*BNC1*	**2.10**	**0.00**	**(1.36-3.25)**	1.26	0.61	(0.52-3.06)	1.93	0.30	(0.55-6.75)	1.69	0.11	(0.88-3.21)
*BRCA1*	0.76	0.44	(0.38-1.52)	0.88	0.82	(0.31-2.52)	0.00	1.00	-	2.42	0.16	(0.70-8.34)
*CDKN2B*	0.80	0.49	(0.42-1.51)	0.79	0.59	(0.33-1.90)	1.18	0.84	(0.25-5.63)	1.82	0.33	(0.55-6.03)
*CHFR*	0.38	0.34	(0.05-2.76)	0.53	0.53	(0.07-3.90)	1.00	-	-	1.00	-	-
*ESR1*	1.21	0.45	(0.74-1.99)	0.89	0.75	(0.44-1.82)	0.68	0.64	(0.14-3.40)	1.27	0.57	(0.56-2.89)
*EYA2*	1.41	0.26	(0.78-2.55)	1.93	0.15	(0.79-4.71)	0.54	0.57	(0.07-4.37)	1.31	0.54	(0.55-3.16)
*GSTP1*	**6.91**	**0.00**	**(2.08-22.96)**	1.00	-	-	^*^	1.00	(0.00- --)	2.33	0.26	(0.54-9.99)
*HIC1*	1.37	0.27	(0.78-2.39)	1.49	0.46	(0.51-4.34)	1.00	-	-	0.92	0.82	(0.45-1.88)
*MEST1v2*	1.45	0.16	(0.86-2.45)	1.97	0.13	(0.81-4.79)	1.88	0.36	(0.49-7.22)	1.21	0.63	(0.56-2.64)
*MGMT*	2.21	0.09	(0.88-5.54)	3.02	0.29	(0.39-23.38)	0.71	0.75	(0.09-5.71)	3.45	0.06	(0.96-12.44)
*MLH1*	**1.85**	**0.04**	**(1.03-3.32)**	1.54	0.49	(0.46-5.18)	0.95	0.94	(0.24-3.70)	1.79	0.15	(0.81-3.96)
*NPTX2*	1.05	0.85	(0.65-1.68)	1.12	0.75	(0.55-2.29)	0.70	0.55	(0.22-2.26)	0.62	0.26	(0.27-1.42)
*NEUROG1*	1.41	0.32	(0.72-2.74)	2.51	0.22	(0.57-11.00)	0.38	0.37	(0.05-3.13)	0.85	0.70	(0.38-1.93)
*RARB*	1.07	0.73	(0.71-1.62)	1.03	0.93	(0.53-1.99)	1.64	0.42	(0.49-5.43)	0.98	0.95	(0.53-1.82)
*RASSF1A*	1.30	0.22	(0.86-1.97)	1.35	0.39	(0.68-2.68)	1.08	0.90	(0.34-3.49)	1.33	0.38	(0.70-2.51)
*SFRP1*	**2.11**	**0.00**	**(1.38-3.23)**	1.60	0.17	(0.82-3.13)	3.50	0.08	(0.86-14.22)	**4.57**	**0.00**	**(2.02-10.34)**
*SFRP2*	0.73	0.17	(0.46-1.14)	**0.31**	**0.01**	**(0.14-0.71)**	2.47	0.28	(0.48-12.86)	1.08	0.81	(0.58-2.02)
*SEPT9v2*	**2.37**	**0.00**	**(1.32-4.27)**	3.37	0.25	(0.43-26.37)	1.00	-	-	1.22	0.55	(0.63-2.38)
*SST*	**1.63**	**0.03**	**(1.06-2.51)**	1.15	0.67	(0.60-2.23)	2.44	0.15	(0.72-8.33)	1.67	0.23	(0.73-3.80)
*TFPI2*	**2.22**	**0.00**	**(1.34-3.68)**	1.39	0.50	(0.53-3.63)	5.48	0.17	(0.50-60.52)	**2.59**	**0.01**	**(1.25-5.39)**
*TAC1*	1.44	0.09	(0.95-2.20)	1.06	0.87	(0.55-2.04)	1.28	0.69	(0.37-4.45)	1.69	0.16	(0.81-3.52)
*VIM*	1.55	0.46	(0.49-4.94)	1.20	0.86	(0.16-8.94)	1.00	-	-	1.89	0.39	(0.45-8.00)
*WNT5A*	**2.32**	**0.03**	**(1.09-4.94)**	3.02	0.29	(0.39-23.38)	**7.05**	**0.05**	**(0.97-51.19)**	1.05	0.91	(0.41-2.72)
*CDKN2A*	1.71	0.22	(0.73-3.97)	**9.24**	**0.05**	**(1.03-82.68)**	1.00	-	-	0.76	0.56	(0.29-1.95)
*PENK*	2.03	0.33	(0.49-8.40)	1.00	-	-	1.00	-	-	0.96	0.95	(0.23-4.02)

Variable analyzed by simple Cox regression analysis.

Bold marks the genes with a statistically significant HR.

HR: Hazard ratio.

CI: Confidence interval.

Note: Stage is in accordance with The American Joint Committee on Cancer stage classification.

*One patients with stage III disease had hypermethylation of *GSTP1.* This patient died only eight days after the diagnosis, resulting in a HR of 19.32x10^16 (p-value = 1) for *GSTP1* hypermethylation in stage III disease.

**Figure 3:**
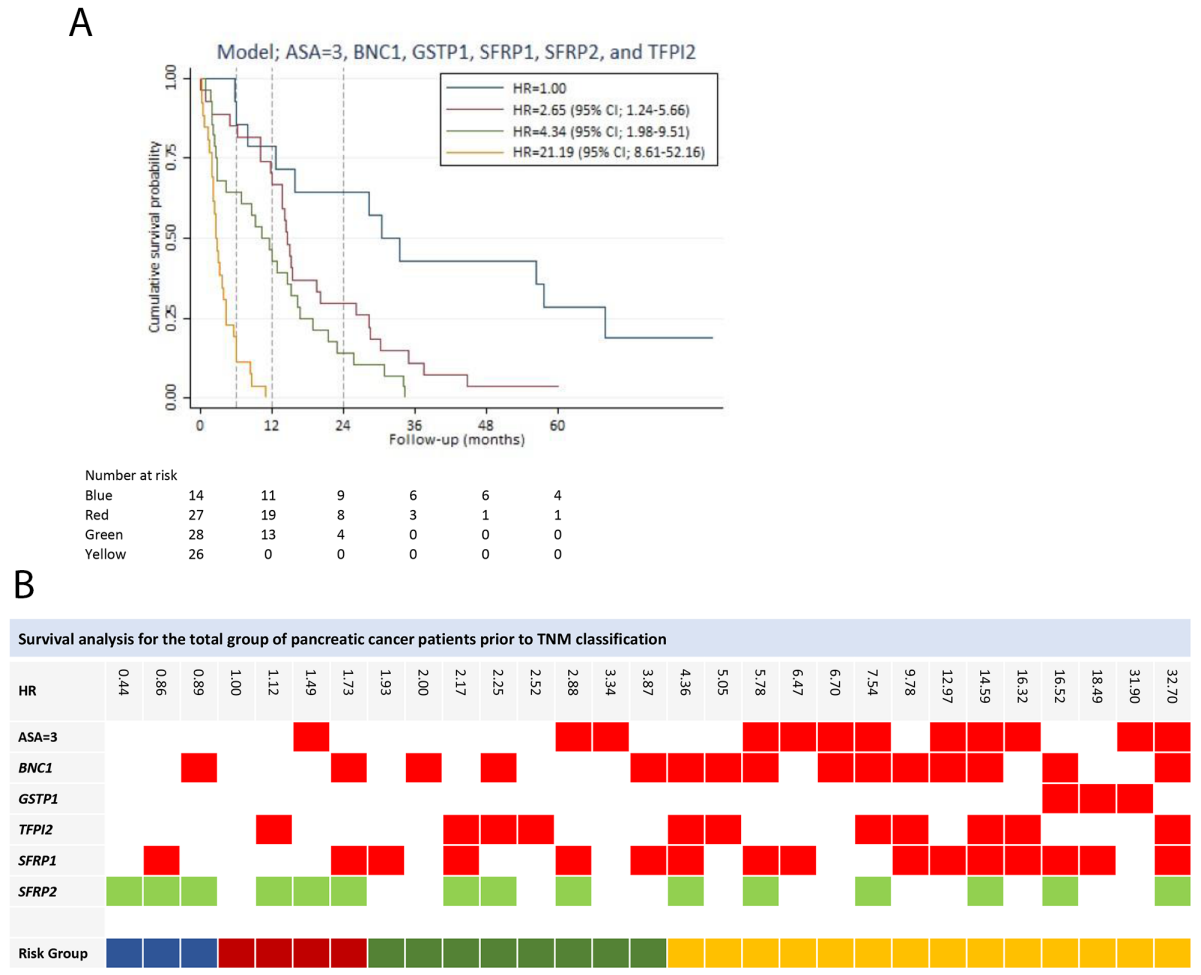
Survival analysis for the total group of patients prior to stage classification **(A)** Survival prediction model for the total group of patients prior to stage classification, developed by multivariable Cox regression analysis using backward stepwise selection. Patients in risk group 2, risk group 3 and risk group 4 had a HR of 2.65 (95% CI; 1.24-5.66), 4.34 (95% CI; 1.98-9.51) and 21.19 (95% CI; 8.61-52.15), respectively, compared to risk group 1. **(B)** The gene combination together with the corresponding the HR is illustrated for the survival prediction model (ASA=3, *BNC1*, *GSTP1*, *TFPI2*, *SFRP1*, and *SFRP2*). Blue: Risk group 1. Red: Risk group 2. Green: Risk group 3. Yellow: Risk group 4. Note: Stage is in accordance with The American Joint Committee on Cancer stage classification.

All of the potential predictors (14 genes out of the 28-gene panel), including an ASA score of three and PS > 0, were used to develop a prognostic prediction model. A model containing the following variables, an ASA score of three, *GSTP1*, *SFRP2*, *BNC1*, *SFRP1*, and *TFPI2*, was determined as the final model with a Harrell’s *c* of 0.73 (Table 3). PS was eliminated in the stepwise selection. Hypermethylation of all the genes in the model yield a HR greater than one, except for *SFRP2* hypermethylation (HR = 0.45 (95% CI; 0.27-0.73)), indicating a positive impact of *SFRP2* hypermethylation on survival. There were no significant interactions between variables in the model, and the model was well calibrated (p-value = 0.9956). The final model had by internal validation an optimism of 0.07, resulting in an optimism corrected Harrell’s *c* of 0.66. Based on the model, patients were divided into four risk groups. The survival curves of the risk groups are illustrated in Figure 3A. The gene combination together with the corresponding HR is shown in Figure 3B.

**Table 3:** Survival prediction models according to stage

	*Harrel’s c*		*ASA=3*	*BMP3*	*BNC1*	*GSTP1*	*MESTv2*	*MGMT*	*NPTX2*	*SFRP1*	*SFRP2*	*TFPI2*
All patients^*^	0.73	*HR 95% CI*	3.34 (1.91-5.84)		2.00 (1.26-3.18)	9.55 (2.70-33.82)				1.94 (1.24-3.02)	0.45 (0.27-0.73)	2.52 (1.42-4.47)
Stage I, II^**^	0.75	*HR 95% CI*	14.13 (4.56-43.81)				2.39 (0.97-5.94)				0.18 (0.07-0.45)	
Stage IV^**^	0.71	*HR 95% CI*		2.65 (1.11-6.29)				2.11 (0.57-7.87)	0.45 (0.17-1.18)	2.77 (1.15-6.67)		

Survival prediction models developed by multivariable Cox regression analysis using backward stepwise selection.

*Survival prediction model for the total group of patients with pancreatic adenocarcinoma without taking stage into account.

**Stage specific survival prediction model.

HR: Hazard ratio

CI: Confidence interval

ASA: American Society of Anesthesiologists score.

Note: Stage is in accordance with The American Joint Committee on Cancer stage classification.

### Prognostic prediction model development for stage I and II pancreatic adenocarcinoma

**Figure 4:**
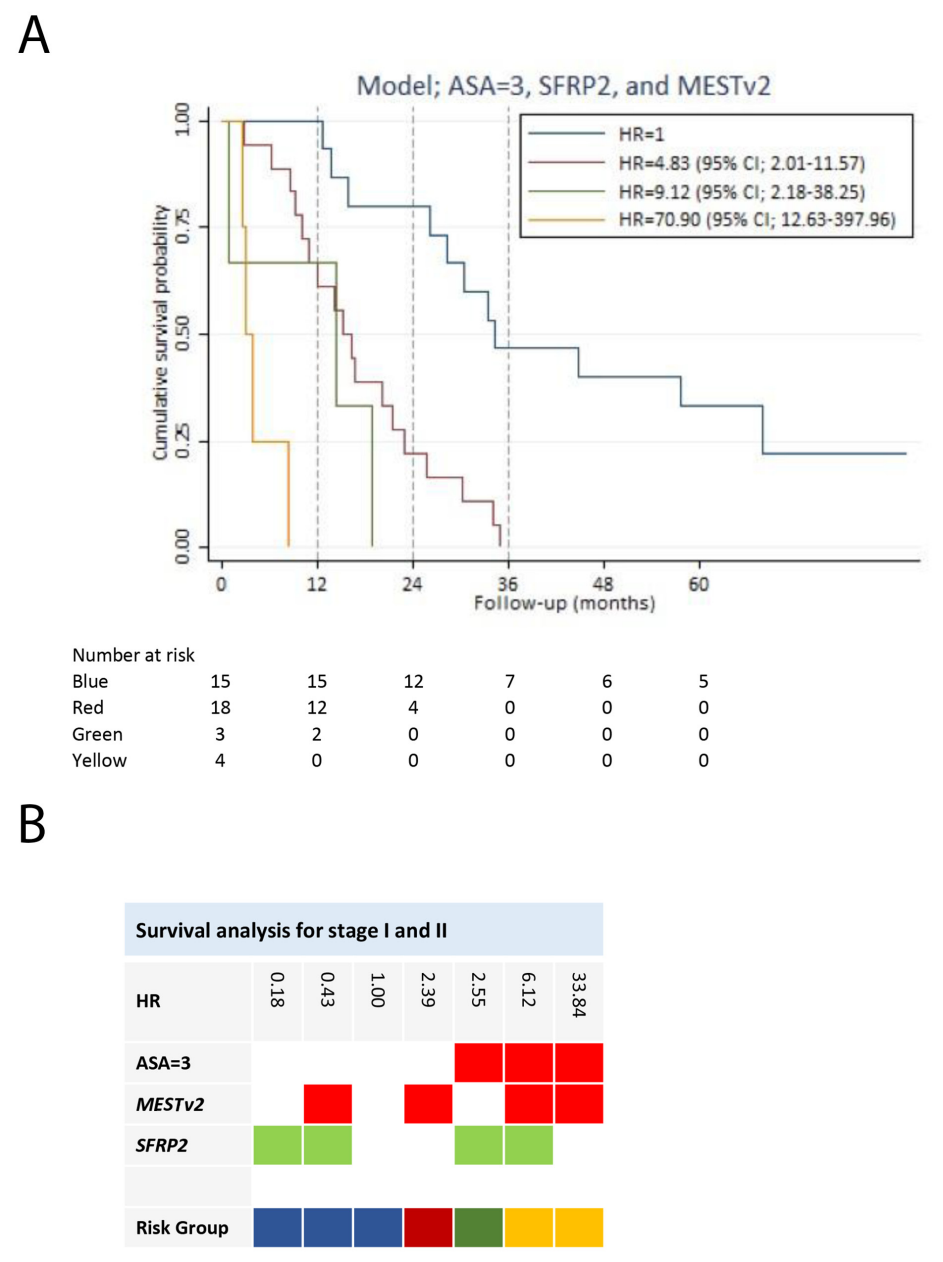
Survival analysis for stage I and II pancreatic adenocarcinoma patients **(A)** Survival prediction model for the stage I and II patients, developed by multivariable Cox regression analysis using backward stepwise selection. Patients in risk group 2, risk group 3 and risk group 4 had a HR of 4.83 (95% CI; 2.01-11.57), 9.12 (95% CI; 2.18-38.25) and 0.90 (95% CI; 12.63-397.96), respectively, compared to risk group 1. **(B)** The gene combination together with the corresponding HR is illustrated for the survival prediction model (ASA=3, *MESTv2*, and *SFRP2*). Blue: Risk group 1. Red: Risk group 2. Green: Risk group 3. Yellow: Risk group 4. Note: Stage is in accordance with The American Joint Committee on Cancer stage classification.

We performed a subgroup analysis of patients with potentially resectable disease. The objective was to develop a survival prediction model for this particular subgroup of patients. Hypermethylation of two genes (*SFRP2* and *CDKN2A*) (Table 2) was, by univariate screening, significantly associated with impaired survival of stage I and II disease, with *CDKN2A* yielding a HR of 9.24 (95% CI; 1.03-82.68). The HRs for age and gender were insignificant. Patients with an ASA score of three compared to an ASA score of one had an increased HR of 4.85 (95% CI; 1.85-12.76). In addition, PS > 0 was associated with a HR of 3.39 (95% CI; 1.64-7.02) compared to PS = 0. In the multivariable analysis, we only included ASA score, as treatment of stage I and II disease primarily is handled by surgeons routinely using ASA score and not PS in the evaluation of operability. Nine hypermethylated genes were potential predictors for survival. These together with an ASA score of three were used to develop a prognostic prediction model for stage I and II pancreatic adenocarcinoma. The final model was an ASA score of three, hypermethylation of *SFRP2* and *MESTv2*, reaching a Harrell’s *c* of 0.75 (Table 3). There were no significant interactions between variables in the model. The final model had by internal validation an optimism of 0.10, resulting in an optimism corrected Harrell’s *c* of 0.65. An ASA score of three was the variable with the greatest negative impact on survival of patients with stage I and II disease (Table 3). *SFRP2* hypermethylation had a highly positive influence on survival (HR = 0.18 (95% CI; 0.07-0.45)), whereas hypermethylation of *MEST1v2* was associated with a negative impact on survival (HR = 2.39 (95% CI; 0.97-5.94)). We divided the patients into four risk groups based on the prediction model for stage I and II disease. Figure 4 illustrates survival of the risk groups. Patients in risk group 1 had a two-year survival of 80% (95% CI; 50%-93%) and a three-year survival of 47% (95% CI; 21%-69%) compared to a two-year survival of only 22% (95% CI; 7%-43%) and none of the patients alive after three years in risk group 2. After five years of follow-up, three patients were alive without residual disease or recurrence. All three patients had an ASA score below three and hypermethylation of *SFRP2* at the time of diagnosis. Patients with an ASA score of three (risk group 3 and risk group 4) had poor survival independent of hypermethylation status (Figure 4).

### Prognostic prediction model development for stage III pancreatic adenocarcinoma

Only hypermethylation of one gene (*WNT5A*) was significantly associated with survival in patients with stage III disease. Due to the limited number of patients in this subgroup, no further analysis was performed.

### Prognostic prediction model development for stage IV pancreatic adenocarcinoma

**Figure 5:**
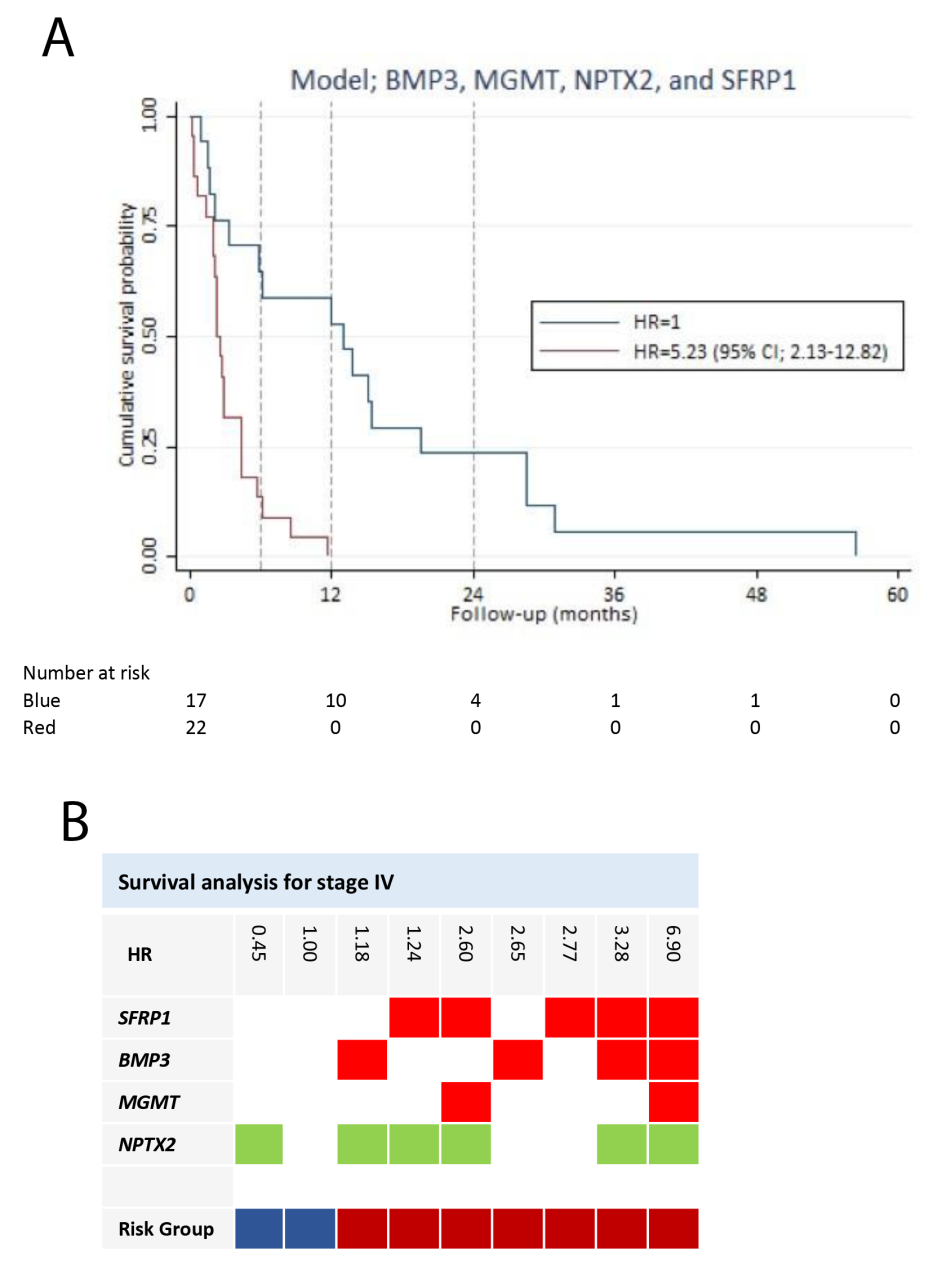
Survival analysis for stage IV pancreatic adenocarcinoma patients **(A)** Survival prediction model for the stage IV patients, developed by multivariable Cox regression analysis using backward stepwise selection. Patients in risk group 2 had a HR of 5.23 (95% CI; 2.13-12.82), compared to patients in risk group 1. **(B)** The gene combination together with the corresponding HR is illustrated for the survival prediction model (*BMP3*, *MGMT*, *NPTX2,* and *SFRP1*). Blue: Risk group 1. Red: Risk group 2. Note: Stage is in accordance with The American Joint Committee on Cancer stage classification.

We performed a subgroup analysis of stage IV disease, with the purpose of developing a model to predict the survival of patients with distant metastases. In the univariate screening, three genes (*BMP3*, *SFRP1*, and *TFPI2*) (Table 2) were associated with a significant increased HR. *SFRP1* yield the greatest HR of 4.57 (95% CI; 2.02-10.34)). The HRs for age, gender and ASA score were insignificant. Patients with a PS > 0 had a HR of 1.78 (95% CI; 0.95-3.36) compared to patients with a PS = 0. PS was excluded from the multivariable analysis, because it was insignificantly associated with survival of stage IV disease (p-value = 0.074). A prognostic model was developed based on hypermethylation of 11 potential predictor genes. The final model (*BMP3*, *MGMT*, *NPTX2*, and *SFRP1*) reached a Harrell’s *c* of 0.71 and was well calibrated (p-value = 0.3517) (Table 3). There were no significant interactions in the model. The final model had by internal validation an optimism of 0.12, resulting in an optimism corrected Harrell’s c of 0.59. All of the variables were associated with impaired survival, except *NPTX2* yielding a HR of 0.45 (95% CI; 0.17-1.18). Based on the prediction model, patients with stage IV disease were divided into two risk groups (Figure 5). Patients in risk group 2 had a HR of 5.23 (95% CI; 2.13-12.82) compared to patients in risk group 1. Patients in risk group 1 had a better 6-month and one-year survival (64% (95% CI; 38%-82%), and 59% (95% CI; 33%-78%), respectively) compared to patients in risk group 2 with a 6-month survival of 14% (95% CI; 3%-30%), and unfortunately, none of the patients were alive after one year (Figure 5).

## DISCUSSION

Promoter hypermethylation of tumor suppressor genes is a hallmark of cancer [11–13]. In the context of pancreatic cancer, aberrant DNA hypermethylation has been detected in cell lines [21], tumor tissue [22], stool [23], pancreatic juice [24– 26] and cell-free DNA [20], [27], [28]. The majority of studies have determined the diagnostic value, and only very few studies have investigated the prognostic value of hypermethylated DNA [29], [30].

In this study, we analyzed promoter hypermethylation of 28 genes in plasma-derived cell-free DNA of patients with pancreatic adenocarcinoma according to survival. We found significantly shorter survival time of patients with more than 10 hypermethylated genes in cell-free DNA, compared to patients with fewer hypermethylated genes. To our knowledge, this has not previously been described with regard to pancreatic cancer. However, a similar finding was described regarding head and neck squamous cell carcinoma, where hypermethylation in tumor tissue of more than six out of 11 genes was associated with poor overall survival [17].

We found that the survival of pancreatic adenocarcinoma patients was associated with hypermethylation of several individual genes, varying with cancer stage.

Hypermethylation of *SFRP1*, *BNC1,* and *TFPI2* were in the univariate screening associated with poor survival for stage IV pancreatic adenocarcinoma. *SFRP1* hypermethylation had the greatest HR. The *SFRP1* gene encodes for secreted frizzled-related protein 1, which acts as a modulator of the Wnt signaling pathway [31]. Upregulation of the Wnt pathway due to promoter hypermethylation of *SFRP* genes has been associated with cancer formation. *SFRP1* promoter hypermethylation has previously been detected in tumor tissue [32], pancreatic juice [24] and cell-free DNA [27] from patients with pancreatic cancer. A prognostic value of *SFRP1* hypermethylation has, to our knowledge, not previously been described regarding pancreatic adenocarcinoma. However, in line with our findings, studies on tumor tissue regarding breast cancer [18] and renal cancer [33] have suggested *SFRP1* hypermethylation to be an independent risk factor for low overall survival.

We also showed that hypermethylation of *BMP3* was associated with decreased survival in stage IV pancreatic adenocarcinoma. The *BMP3* gene encodes methylated bone morphogenetic protein 3, related to the TGF-beta pathway. [34]. Previous studies on cholangiocarcinoma [35] and colorectal cancer [36] have suggested a tumor suppressor function of *BMP3*. In addition, studies have indicated that *BMP3* hypermethylation has diagnostic value in stool from patients with pancreatic cancer [23] and colorectal cancer [23], [37]. To our knowledge, we are the first to describe a prognostic value of *BMP3* hypermethylation with regard to stage IV pancreatic adenocarcinoma.

Furthermore, our study indicates that *TFPI2* hypermethylation has a negative impact on survival in stage IV disease. The *TFPI2* gene encodes for tissue factor pathway inhibitor 2 protein, which is associated with cell adhesion and the clotting cascade [38]. The gene has been identified as a tumor suppressor gene in several types of cancer, where promoter hypermethylation has been the cause of gene silencing [39–41]. *TFPI2* hypermethylation has been detected in tissue from intraductal papillary mucinous neoplasms [42], in pancreatic cancer tissue [43] and in pancreatic juice [26] from pancreatic cancer patients. The prognostic value of *TFPI2* hypermethylation has not previously been evaluated in pancreatic cancer. However, consistent with our finding, *TFPI2* hypermethylation in tissue from hepatocellular carcinoma has been found to correlate with advanced cancer stage and a significantly shorter survival time [39]. Furthermore, hypermethylation of *TFPI2* in the serum of melanoma patients has been suggested as a biomarker for metastatic disease [41].

Our study suggests that hypermethylation of *SFRP2* has a positive impact on survival in stage I and II pancreatic adenocarcinoma. The *SFRP2* gene, which like *SFRP1*, modulates the Wnt signaling pathway [31]. Hypermethylation of *SFRP2* has previously been associated with the development of colorectal cancer [44–46], gastric cancer [47], and pancreatic cancer [20], [23], [32]. However, to our knowledge, hypermethylation of *SFRP2* in cell-free DNA has not previously been associated with improved prognosis.

Based on the 28-gene panel, we developed prognostic prediction models, which enabled us to stratify patients into risk groups according to survival. We developed a prognostic prediction model based on the total group of patients with pancreatic adenocarcinoma, without considering stage classification. In addition, we developed stage specific prognostic prediction model that further our knowledge of disease aggressiveness within each cancer stage. ASA score was significant in the model for early stage cancer. This finding is consistent with the survival of patients with early stage pancreatic cancer being dependent on surgical treatment, as patients with a high ASA score are more likely not to overcome extensive surgery [48]. Both of the cancer stage-specific prediction models contained a hypermethylated gene with a positive impact on survival. *SFRP2* hypermethylation had a positive influence on survival of stage I and II pancreatic adenocarcinoma. We showed a similar positive trend for *NPTX2* hypermethylation in stage IV disease. The *NPTX2* gene encodes for the neuronal pentraxin 2 protein [49]. Previous studies have shown a diagnostic value of *NPTX2* hypermethylation in pancreatic cancer [27], [50]. In contrast to our finding, hypermethylation of *NPTX2* has been correlated with poor survival in glioblastoma [51]. The conflicting results might reflect a tissue-specific response or result from different parts of the promoter sequence being analyzed. Furthermore, it may suggest that the impact of *NPTX2* hypermethylation differs according to cancer stage.

There is evidence that hypermethylation in cell-free DNA reflects the tumor biology and the heterogeneity of pancreatic cancer [52]. Our study indicates a biological variation in tumors that influences patient outcome and prognosis. Overall, hypermethylation has a negative impact on the survival. However, hypermethylation of a few specific genes seems to have a positive impact. Our findings are consistent with a study by Thomson et al. on pancreatic adenocarcinoma tissue, describing a “survival-” methylation signature associated with short survival time and a “survival+” methylation signature associated with a long survival time [53]. Two previous studies also managed to stratify patients in a low-risk and a high-risk group based on the gene expression profile in pancreatic adenocarcinoma tissue [54],[55]. Our prediction models also enabled stratification of patients in risk groups according to survival. However, the previously described prognostic studies are all tissue-based. Our prognostic prediction models are blood-based tests. Blood-based markers have several advantages compared to tissue-based markers, as retrieving plasma is a minimally invasive procedure without discomfort or risk of complications. In addition, representative tissue samples from small pancreatic tumors can be difficult to obtain [56]. Our survival prediction models have the potential to work as prognostic markers as a supplement to existing clinical tools, which clearly would benefit patients and facilitate tailored treatment.

Our study has some limitations. The study was exploratory, only analyzing a single group of patients. The finding need to be interpreted very carefully. External validation in an independent cohort is required to verify the result and is considered the gold standard for biomarker validation. However, it was impossible for us to reach this standard during the development phase, as pancreatic adenocarcinoma is a relatively rare disease. Therefore internal validation using a bootstrap model was performed, which revealed a relatively large optimism of the prediction models. This most likely reflects the fact that the stage-specific subgroups only contained a limited number of patients, which definitely is a significant limitation of this current study. Larger subgroups would have improved the power of the study and might enabled detection of genes significantly associated with the survival of stage III pancreatic adenocarcinoma.

For methylation analysis, we performed bisulfite treatment followed by a first and second round of methylation-specific PCR, which is a quantitative method [57]. However, we analyzed hypermethylation as a binary variable after dichotomization, as the study lacked sufficient power to conduct a quantitative statistical analysis. Furthermore, the method we used did not provide information regarding the numbers or proportion of methylated CpGs methylated in the investigated part of the promoter sequence. Detailed information about methylated CpGs could have been achieved by DNA sequencing of the PCR products. Unfortunately, this was not possible in our set up [58].

## MATERIALS AND METHODS

### Study design

The study was conducted as a prospective observational cohort study of patients with pancreatic adenocarcinoma, who were admitted to the Department of Gastrointestinal Surgery, Aalborg University Hospital, from February 2008 until February 2011 [59]. All of the participants gave written informed consent. The study was registered in ClinicalTrails.gov: NCT02079363 and approved by the Research Ethics Committee for the North Denmark Region (N-2013037).

### Participants

Consecutive patients with pancreatic adenocarcinoma were included prospectively [59]. Blood samples were collected on admission before the diagnostic work-up and treatment. WHO performance status (PS) and American Society of Anesthesiologists (ASA) scores were registered at inclusion. Patients were excluded if they had concomitant or previous cancer (within three years), previous venous thromboembolism, ongoing anticoagulant, known congenital thrombophilia or connective tissue disease. Patients were followed for five years. Data from the same patients were used in a previous study [20].

### Diagnosis and stage classification

Computed tomography (CT) and positron emission tomography (PET) scans of the thorax and abdomen were performed in the diagnostic work-up of all the patients. The cancer diagnosis was confirmed by histopathological analysis of biopsy specimens obtained by percutaneous, endoscopic or laparoscopic ultrasound. Patients were staged according to TNM classification 7^th^ Edition [3]. The T and N categories were determined by histopathological analysis for patients who underwent intended curative surgery. If surgery was not performed, the final clinical decision determined the TNM stage. Consensus was achieved for staging and treatment of all the patients at multidisciplinary team conferences [59].

### Blood sampling and analytical method

Skilled technicians obtained the blood samples by peripheral venipuncture [60]. EDTA plasma was centrifuged for 20 min (4000 rpm) at 4°C and stored within two hours after sampling in a biobank at -80°C until further methylation analysis [20].

A single skilled laboratory scientist performed all of the methylation analyses. The analyses were performed blinded. Extraction and deamination of cell-free DNA was performed as previously described by our group [20], [57].

To expand the amount of relevant deaminated DNA, a first round PCR amplification was performed with a mix of methylation-specific outer primers for all of the investigated promoter regions. Thereafter, a second round of PCR was performed, using inner methylation-specific primers and methylation-specific probes in individual reactions for each investigated promoter region. The primer and probe sequences is previously described [20].

We analyzed a panel of 28 selected genes. The selection of genes has been described previously by our group [20]. Hemi-methylated *MEST transcript variant 1* was used as a reference gene in both the first and second round PCR. The selected panel of genes was previously tested as diagnostic markers for pancreatic adenocarcinoma [20].

### Outcome

The primary outcome of the study was overall survival of patients with pancreatic adenocarcinoma. Survival time was calculated as the difference between date of inclusion in the study (the date the patient was referred to the hospital suspected of or with symptoms of pancreatic cancer) and the date of censoring/ date of death.

### Statistical analysis methods

We analyzed each gene in the panel as binary variables after a dichotomization. A threshold cycle (Ct) of zero was interpreted as a non-methylated gene, and a Ct above zero was interpreted as a hypermethylated gene.

Patients were divided into groups according to the TNM classification [3]. Survival according to stage was assessed using Kaplan-Meier survival curves.

For each patient, the total number of hypermethylated genes was calculated, and based on those calculations, patients were divided into quartiles. Survival according to the total number of hypermethylated genes was evaluated using Kaplan-Meier survival curves.

Survival analysis was performed using Cox proportional hazards regression as described below for the total patient group and for subgroups according to cancer stage ((I and II), (III) and (IV)). Unless otherwise stated, a p-value < 0.05 was considered statistically significant.

### Survival prediction model development

*1. Screening of each individual variable as a predictor of survival:* Cox regression was performed for each gene in the panel and for age > 65, gender, PS and ASA score. The hazard ratios (HR) and p-values were calculated. Variables with a p-value < 0.3 were considered as potential predictors and selected for further analysis.

*2. Variable selection:* Stepwise backward elimination in Cox regression models was performed to select the relevant variables using 0.05 as the significance level for removal from the model. For each intermediate model, Harrell’s overall concordance (*c*) statistic was calculated [61].

*3. Determination of the best model:* The model with the best performance measure according to Harrell’s *c* was determined as the final model.

*4. Interactions between the variables*: The interaction between all of the variables was assessed in the final models. Interactions with a p-value < 0.01 were considered statistically significant.

*5. Validation:* The May-Hosmer goodness-of-fit test was performed for calibration performance. Bootstrap procedure was used for internal validation of the models.

Patients were divided into risk-groups with regard to the final survival prediction models.

The risk groups for each survival prediction model serve for illustration purposes only and were defined as follows:

- For each gene combination observed in the data the hazard ratio was calculated and Kaplan-Meier survival curves were used to illustrate the corresponding survival.

- Patients were stratified into risk groups according to their gene combination based on what visually seemed as the most natural grouping of the survival curves of each gene combination.

- Finally, Kaplan-Meier survival curves were used to illustrate the survival according to the defined risk-groups of each survival prediction model.

All of the data were analyzed using STATA 14.0 software [StataCorp LP, Texas].

All of the authors had full access to the study data, reviewed and approved the final manuscript.

## CONCLUSION

We found that hypermethylation of more than 10 genes in plasma-derived cell-free DNA is an independent risk factor of poor overall survival in patients with pancreatic adenocarcinoma. Furthermore, survival of pancreatic adenocarcinoma patients is associated with promoter hypermethylation of several specific genes, varying with cancer stage. Prediction models based on cell-free DNA hypermethylation enabled us to stratify pancreatic adenocarcinoma patients into risk groups according to survival. The models have the potential to provide additional information to the TNM classification as prognostic biomarkers and thereby facilitate tailored treatment. Furthermore, genes in these models, or the pathways they are involved in, may represent future therapeutic targets. Validation of the finding is, however, required to substantiate the results.

## References

[1] Siegel R, Ma J, Zou Z, Jemal A (2014). Cancer statistics, 2014. CA Cancer J Clin.

[2] “American Cancer Society: Pancreatic cancer.” Online materiel, http://www.cancer.org/cancer/pancreaticcancer (Accessed December 2016)

[3] “American Joint Committee on Cancer, Pancreas Cancer Staging, 7th Edition.” Online materiel, http://www.cancerstaging.org/references-tools/quickre (Accessed December 2016)

[4] Bailey P, Chang DK, Nones K, Johns AL, Patch AM, Gingras MC, Miller DK, Christ AN, Bruxner TJ, Quinn MC, Nourse C, Murtaugh LC, Harliwong I (2016). Genomic analyses identify molecular subtypes of pancreatic cancer. Nature.

[5] Kondo N, Murakami Y, Uemura K, Hayashidani Y, Sudo T, Hashimoto Y, Nakashima A, Sakabe R, Shigemoto N, Kato Y, Ohge H, Sueda T (2010). Prognostic impact of perioperative serum CA 19-9 levels in patients with resectable pancreatic cancer. Ann Surg Oncol.

[6] Reni M, Cereda S, Balzano G, Passoni P, Rognone A, Fugazza C, Mazza E, Zerbi A, Di Carlo V, Villa E (2009). Carbohydrate antigen 19-9 change during chemotherapy for advanced pancreatic adenocarcinoma. Cancer.

[7] Louvet C, Labianca R, Hammel P, Lledo G, Zampino MG, André T, Zaniboni A, Ducreux M, Aitini E, Taïeb J, Faroux R, Lepere C, de Gramont A, GERCOR, and GISCAD (2005). Gemcitabine in combination with oxaliplatin compared with gemcitabine alone in locally advanced or metastatic pancreatic cancer: results of a GERCOR and GISCAD phase III trial. J Clin Oncol.

[8] Park JK, Yoon YB, Kim YT, Ryu JK, Yoon WJ, Lee SH (2008). Survival and prognostic factors of unresectable pancreatic cancer. J Clin Gastroenterol.

[9] Delpu Y, Hanoun N, Lulka H, Sicard F, Selves J, Buscail L, Torrisani J, Cordelier P (2011). Genetic and epigenetic alterations in pancreatic carcinogenesis. Curr Genomics.

[10] Lomberk GA (2011). Epigenetic silencing of tumor suppressor genes in pancreatic cancer. J Gastrointest Cancer.

[11] Lomberk G, Mathison AJ, Grzenda A, Urrutia R (2008). The sunset of somatic genetics and the dawn of epigenetics: a new frontier in pancreatic cancer research. Curr Opin Gastroenterol.

[12] Mulero-Navarro S, Esteller M (2008). Epigenetic biomarkers for human cancer: the time is now. Crit Rev Oncol Hematol.

[13] Costa FF (2010). Epigenomics in cancer management. Cancer Manag Res.

[14] Sebova K, Fridrichova I (2010). Epigenetic tools in potential anticancer therapy. Anticancer Drugs.

[15] Philipp AB, Stieber P, Nagel D, Neumann J, Spelsberg F, Jung A, Lamerz R, Herbst A, Kolligs FT (2012). Prognostic role of methylated free circulating DNA in colorectal cancer. Int J Cancer.

[16] Wallner M, Herbst A, Behrens A, Crispin A, Stieber P, Göke B, Lamerz R, Kolligs FT (2006). Methylation of serum DNA is an independent prognostic marker in colorectal cancer. Clin Cancer Res.

[17] Misawa K, Mochizuki D, Imai A, Endo S, Mima M, Misawa Y, Kanazawa T, Carey TE, Mineta H (2016). Prognostic value of aberrant promoter hypermethylation of tumor-related genes in early-stage head and neck cancer. Oncotarget.

[18] Veeck J, Niederacher D, An H, Klopocki E, Wiesmann F, Betz B, Galm O, Camara O, Dürst M, Kristiansen G, Huszka C, Knüchel R, Dahl E (2006). Aberrant methylation of the Wnt antagonist SFRP1 in breast cancer is associated with unfavourable prognosis. Oncogene.

[19] Henriksen SD, Madsen PH, Krarup H, Thorlacius-Ussing O (2015). DNA Hypermethylation as a Blood-Based Marker for Pancreatic Cancer: A Literature Review. Pancreas.

[20] Henriksen SD, Madsen PH, Larsen AC, Johansen MB, Drewes AM, Pedersen IS, Krarup H, Thorlacius-Ussing O (2016). Cell-free DNA promoter hypermethylation in plasma as a diagnostic marker for pancreatic adenocarcinoma. Clin Epigenetics.

[21] Ueki T, Toyota M, Skinner H, Walter KM, Yeo CJ, Issa JP, Hruban RH, Goggins M (2001). Identification and characterization of differentially methylated CpG islands in pancreatic carcinoma. Cancer Res.

[22] Ueki T, Toyota M, Sohn T, Yeo CJ, Issa JP, Hruban RH, Goggins M (2000). Hypermethylation of multiple genes in pancreatic adenocarcinoma. Cancer Res.

[23] Kisiel JB, Yab TC, Taylor WR, Chari ST, Petersen GM, Mahoney DW, Ahlquist DA (2012). Stool DNA testing for the detection of pancreatic cancer: assessment of methylation marker candidates. Cancer.

[24] Watanabe H, Okada G, Ohtsubo K, Yao F, Jiang PH, Mouri H, Wakabayashi T, Sawabu N (2006). Aberrant methylation of secreted apoptosis-related protein 2 (SARP2) in pure pancreatic juice in diagnosis of pancreatic neoplasms. Pancreas.

[25] Yao F, Sun M, Dong M, Jing F, Chen B, Xu H, Wang S (2013). NPTX2 hypermethylation in pure pancreatic juice predicts pancreatic neoplasms. Am J Med Sci.

[26] Matsubayashi H, Canto M, Sato N, Klein A, Abe T, Yamashita K, Yeo CJ, Kalloo A, Hruban R, Goggins M (2006). DNA methylation alterations in the pancreatic juice of patients with suspected pancreatic disease. Cancer Res.

[27] Park JW, Baek IH, Kim YT (2012). Preliminary study analyzing the methylated genes in the plasma of patients with pancreatic cancer. Scand J Surg.

[28] Liggett T, Melnikov A, Yi QL, Replogle C, Brand R, Kaul K, Talamonti M, Abrams RA, Levenson V (2010). Differential methylation of cell-free circulating DNA among patients with pancreatic cancer versus chronic pancreatitis. Cancer.

[29] Ohtsubo K, Watanabe H, Yamaguchi Y, Hu YX, Motoo Y, Okai T, Sawabu N (2003). Abnormalities of tumor suppressor gene p16 in pancreatic carcinoma: immunohistochemical and genetic findings compared with clinicopathological parameters. J Gastroenterol.

[30] Zhou YF, Xu W, Wang X, Sun JS, Xiang JJ, Li ZS, Zhang XF (2014). Negative methylation status of vimentin predicts improved prognosis in pancreatic carcinoma. World J Gastroenterol.

[31] Kawano Y, Kypta R (2003). Secreted antagonists of the Wnt signalling pathway. J Cell Sci.

[32] Bu XM, Zhao CH, Zhang N, Gao F, Lin S, Dai XW (2008). Hypermethylation and aberrant expression of secreted frizzled-related protein genes in pancreatic cancer. World J Gastroenterol.

[33] Ricketts CJ, Hill VK, Linehan WM (2014). Tumor-specific hypermethylation of epigenetic biomarkers, including SFRP1, predicts for poorer survival in patients from the TCGA Kidney Renal Clear Cell Carcinoma (KIRC) project. PLoS One.

[34] Ducy P, Karsenty G (2000). The family of bone morphogenetic proteins. Kidney Int.

[35] Kisiel JB, Li J, Zou H, Oseini AM, Strauss BB, Gulaid KH, Moser CD, Aderca I, Ahlquist DA, Roberts LR, Shire AM (2013). Methylated Bone Morphogenetic Protein 3 (BMP3) Gene: Evaluation of Tumor Suppressor Function and Biomarker Potential in Biliary Cancer. J Mol Biomark Diagn.

[36] Loh K, Chia JA, Greco S, Cozzi SJ, Buttenshaw RL, Bond CE, Simms LA, Pike T, Young JP, Jass JR, Spring KJ, Leggett BA, Whitehall VL (2008). Bone morphogenic protein 3 inactivation is an early and frequent event in colorectal cancer development. Genes Chromosomes Cancer.

[37] Imperiale TF, Ransohoff DF, Itzkowitz SH, Levin TR, Lavin P, Lidgard GP, Ahlquist DA, Berger BM (2014). Multitarget stool DNA testing for colorectal-cancer screening. N Engl J Med.

[38] Sprecher CA, Kisiel W, Mathewes S, Foster DC (1994). Molecular cloning, expression, and partial characterization of a second human tissue-factor-pathway inhibitor. Proc Natl Acad Sci USA.

[39] Sun FK, Sun Q, Fan YC, Gao S, Zhao J, Li F, Jia YB, Liu C, Wang LY, Li XY, Ji XF, Wang K (2016). Methylation of tissue factor pathway inhibitor 2 as a prognostic biomarker for hepatocellular carcinoma after hepatectomy. J Gastroenterol Hepatol.

[40] Hibi K, Goto T, Shirahata A, Saito M, Kigawa G, Nemoto H, Sanada Y (2011). Detection of TFPI2 methylation in the serum of colorectal cancer patients. Cancer Lett.

[41] Lo Nigro C, Wang H, McHugh A, Lattanzio L, Matin R, Harwood C, Syed N, Hatzimichael E, Briasoulis E, Merlano M, Evans A, Thompson A, Leigh I (2013). Methylated tissue factor pathway inhibitor 2 (TFPI2) DNA in serum is a biomarker of metastatic melanoma. J Invest Dermatol.

[42] Hong SM, Kelly D, Griffith M, Omura N, Li A, Li CP, Hruban RH, Goggins M (2008). Multiple genes are hypermethylated in intraductal papillary mucinous neoplasms of the pancreas. Mod Pathol.

[43] Sato N, Parker AR, Fukushima N, Miyagi Y, Iacobuzio-Donahue CA, Eshleman JR, Goggins M (2005). Epigenetic inactivation of TFPI-2 as a common mechanism associated with growth and invasion of pancreatic ductal adenocarcinoma. Oncogene.

[44] Zhang X, Song YF, Lu HN, Wang DP, Zhang XS, Huang SL, Sun BL, Huang ZG (2015). Combined detection of plasma GATA5 and SFRP2 methylation is a valid noninvasive biomarker for colorectal cancer and adenomas. World J Gastroenterol.

[45] Lu H, Huang S, Zhang X, Wang D, Zhang X, Yuan X, Zhang Q, Huang Z (2014). DNA methylation analysis of SFRP2, GATA4/5, NDRG4 and VIM for the detection of colorectal cancer in fecal DNA. Oncol Lett.

[46] Silva AL, Dawson SN, Arends MJ, Guttula K, Hall N, Cameron EA, Huang TH, Brenton JD, Tavaré S, Bienz M, Ibrahim AE (2014). Boosting Wnt activity during colorectal cancer progression through selective hypermethylation of Wnt signaling antagonists. BMC Cancer.

[47] Zhang X, Zhang X, Sun B, Lu H, Wang D, Yuan X, Huang Z (2014). Detection of aberrant promoter methylation of RNF180, DAPK1 and SFRP2 in plasma DNA of patients with gastric cancer. Oncol Lett.

[48] Wiltberger G, Muhl B, Benzing C, Atanasov G, Hau HM, Horn M, Krenzien F, Bartels M (2016). Preoperative risk stratification for major complications following pancreaticoduodenectomy: identification of high-risk patients. Int J Surg.

[49] Hsu YC, Perin MS (1995). Human neuronal pentraxin II (NPTX2): conservation, genomic structure, and chromosomal localization. Genomics.

[50] Park JK, Ryu JK, Lee KH, Lee JK, Yoon WJ, Lee SH, Yoo JW, Woo SM, Lee GY, Lee CH, Kim YT, Yoon YB (2007). Quantitative analysis of NPTX2 hypermethylation is a promising molecular diagnostic marker for pancreatic cancer. Pancreas.

[51] Shukla S, Pia Patric IR, Thinagararjan S, Srinivasan S, Mondal B, Hegde AS, Chandramouli BA, Santosh V, Arivazhagan A, Somasundaram K (2013). A DNA methylation prognostic signature of glioblastoma: identification of NPTX2-PTEN-NF-κB nexus. Cancer Res.

[52] Yi JM, Guzzetta AA, Bailey VJ, Downing SR, Van Neste L, Chiappinelli KB, Keeley BP, Stark A, Herrera A, Wolfgang C, Pappou EP, Iacobuzio-Donahue CA, Goggins MG (2013). Novel methylation biomarker panel for the early detection of pancreatic cancer. Clin Cancer Res.

[53] Thompson MJ, Rubbi L, Dawson DW, Donahue TR, Pellegrini M (2015). Pancreatic cancer patient survival correlates with DNA methylation of pancreas development genes. PLoS One.

[54] Stratford JK, Bentrem DJ, Anderson JM, Fan C, Volmar KA, Marron JS, Routh ED, Caskey LS, Samuel JC, Der CJ, Thorne LB, Calvo BF, Kim HJ (2010). A six-gene signature predicts survival of patients with localized pancreatic ductal adenocarcinoma. PLoS Med.

[55] Newhook TE, Blais EM, Lindberg JM, Adair SJ, Xin W, Lee JK, Papin JA, Parsons JT, Bauer TW (2014). A thirteen-gene expression signature predicts survival of patients with pancreatic cancer and identifies new genes of interest. PLoS One.

[56] Volmar KE, Vollmer RT, Jowell PS, Nelson RC, Xie HB (2005). Pancreatic FNA in 1000 cases: a comparison of imaging modalities. Gastrointest Endosc.

[57] Pedersen IS, Krarup HB, Thorlacius-Ussing O, Madsen PH (2012). High recovery of cell-free methylated DNA based on a rapid bisulfite-treatment protocol. BMC Mol Biol.

[58] Kurdyukov S, Bullock M (2016). DNA Methylation Analysis: Choosing the Right Method. Biology (Basel).

[59] Larsen AC, Dabrowski T, Frøkjær JB, Fisker RV, Iyer VV, Møller BK, Kristensen SR, Thorlacius-Ussing O (2014). Prevalence of venous thromboembolism at diagnosis of upper gastrointestinal cancer. Br J Surg.

[60] Jespersen J, Bertina R, Haverkate F (1999). Laboratory Techniques in Thrombosis - A manual: Second Revised Edition of the Ecat Assay Procedures.

[61] Harrell FE, Lee KL, Mark DB (1996). Multivariable prognostic models: issues in developing models, evaluating assumptions and adequacy, and measuring and reducing errors. Stat Med.

